# Design and Application of Hybrid Polymer-Protein Systems in Cancer Therapy

**DOI:** 10.3390/polym15092219

**Published:** 2023-05-08

**Authors:** Qi Sun, Zhenzhen Yang, Xianrong Qi

**Affiliations:** 1 Department of Pharmaceutics, School of Pharmaceutical Sciences, Capital Medical University, Beijing 100069, China; 2 Beijing Area Major Laboratory of Peptide and Small Molecular Drugs, Beijing 100069, China; 3 Engineering Research Center of Endogenous Prophylactic of Ministry of Education of China, Beijing 100069, China; 4 Drug Clinical Trial Center, Peking University Third Hospital, Peking University, Beijing 100191, China; 5 Institute of Medical Innovation and Research, Peking University Third Hospital, Beijing 100191, China; 6 Department of Pharmaceutics, School of Pharmaceutical Sciences, Peking University, Beijing 100191, China

**Keywords:** polymer, protein, cancer therapy, design, application

## Abstract

Polymer-protein systems have excellent characteristics, such as non-toxic, non-irritating, good water solubility and biocompatibility, which makes them very appealing as cancer therapeutics agents. Inspiringly, they can achieve sustained release and targeted delivery of drugs, greatly improving the effect of cancer therapy and reducing side effects. However, many challenges, such as reducing the toxicity of materials, protecting the activities of proteins and controlling the release of proteins, still need to be overcome. In this review, the design of hybrid polymer–protein systems, including the selection of polymers and the bonding forms of polymer–protein systems, is presented. Meanwhile, vital considerations, including reaction conditions and the release of proteins in the design process, are addressed. Then, hybrid polymer–protein systems developed in the past decades for cancer therapy, including targeted therapy, gene therapy, phototherapy, immunotherapy and vaccine therapy, are summarized. Furthermore, challenges for the hybrid polymer–protein systems in cancer therapy are exemplified, and the perspectives of the field are covered.

## 1. Introduction

In 2020, 19.3 million new cancer cases and almost 10.0 million cancer deaths occurred worldwide [1]. According to the National Cancer Institute (NCI), cancer is predicted to be the leading cause of death from noncommunicable diseases worldwide and a major global health issue. Although we have made incredible progress in the fight against cancer, improving drug bioavailability and efficacy and reducing adverse side effects have always been the focus of research in the field of cancer treatment.

Proteins are biomacromolecules widely present in biological processes in vivo with important functions, such as biological catalysis, high-affinity molecule recognition, and activation and/or inhibition of cellular pathways [2]. Moreover, because of their endogenous nature, proteins are biocompatible and biodegradable. Equally important, their degradation products have been known and eliminated by the body, so potential toxicities could be reduced during treatments [3]. However, free protein therapeutics usually have a rapid blood clearance rate and are very susceptible to degradation under changes in pH, temperature, the concentration of certain ions, etc. Moreover, proteins would fold and assemble into specific three-dimensional structures for the enzyme’s active site and exert protein function. In addition, the specific structures are easy to disrupt by typical synthesis reactions and reduce enzymatic activity. Thus, it is necessary to stabilize protein molecules and prevent protein degradation for application.

In recent years, drug delivery systems have always been a research hotspot in cancer therapy. Compared to traditional drug delivery, polymer drug delivery systems can achieve sustained release, targeted delivery of drugs and the functionality to cross biological barriers, greatly improving the effect of treatment and reducing side effects. In addition, polymers extend the range of available drugs, such as protein drugs, peptide drugs, nucleic acid drugs and others [4]. Polymers not only prevent enzymatic degradation of conjugated or encapsulated drugs and clearance from the body’s immune system but also exhibit excellent biocompatibility [5]. These advantages of polymers and proteins make polymer–protein systems useful in cancer therapy.

PEGylation is a representative example of polymer–protein system by attaching polyethylene glycol (PEG) to a protein. In 1977, Davis and his colleagues first demonstrated the binding of monomethoxy-polyethylene glycol (mPEG) to bovine serum albumin (BSA) and bovine liver catalase by covalent attachment using 2,4,6-trichloro-s-triazine (cyanuric chloride) as the coupling agent. Just as they expected, after PEG modification, the immunogenicity of BSA and bovine liver catalase was greatly reduced and their half life in vivo was prolonged [6,7]. They, then, modified more proteins with PEG all of which showed decreased immunogenicity and increased half life in vivo. In 1990, the United States Food and Drug Administration (FDA) approved Adagen (pegademase bovine), the world’s first PEGylated drug, for the treatment of severe combined immunodeficiency disease (SCID) associated with a deficiency of adenosine deaminase. Then, active polymerization atom transfer radical polymerization (ATRP) [8] and reversible addition-fragmentation chain transfer (RAFT) [9] were developed as facile and versatile radical polymerization techniques in 1995 and 1998, respectively. In 2002, the FDA approved Neulasta (pegfilgrastim), a PEGylated granulocyte colony-stimulating factor (PEG-G-CSF) injection developed by Amgen, which is used to treat a deficiency of white blood cells caused by cancer chemotherapy and some other cancers [10]. In 2011, PegIFN, a modified version of the previously approved interferon indicated for the adjuvant, was approved for the treatment of node-positive melanoma [11]. In addition, PegIntron (Schering-Plough, Kenilworth, NJ, USA) and Pegasys (Hoffmann-La Roche, South San Francisco, CA, USA) for the treatment of chronic-hepatitis-C (CHC), and Mircera (Hoffmann-La Roche, South San Francisco, CA, USA) indicated for the treatment of symptomatic anemia associated with chronic kidney disease (CKD) in adult patients approved by the FDA are also typical PEG-modified drugs for clinical treatment of diseases [12,13]. The clinical success of these PEGylated drugs greatly facilitates the translation of novel protein–polymer systems.

However, there are some challenges to the application of polymer–protein systems, such as rapid clearance and low physical stability. Currently, FDA-approved protein conjugates are covalently linked to PEG [14]. These PEGylated protein drugs have a longer half life in the blood and are administered less frequently, showing significant benefits for patients. Even so, PEG still has a number of potential drawbacks that require the development of alternatives, so the development of polymers with enhanced pharmacokinetic properties as well as other advantages, such as improved stability or degradability, is still important to advance the field of polymer–protein systems in cancer treatments.

Recently, more and more new techniques are being used in cancer treatments research, for example, an increasing number of nanotechnology-supported polymeric nanomaterials and protein-based nanomaterials are being studied in vitro and in vivo for cancer therapy, such as chemotherapy, phototherapy, gene therapy, combination therapy and theranostics [15]. In addition, three-dimensional (3D) bioprinting technology-supported functional polymer materials can be used to build tumor models, so cancer therapeutics and drug screening can be better achieved by these new 3D bio-printed models [16]. These intelligent systems have been built employing a variety of fabrication, preparation processes and materials selection to overcome the constraints of single-component systems.

**Figure 1:**
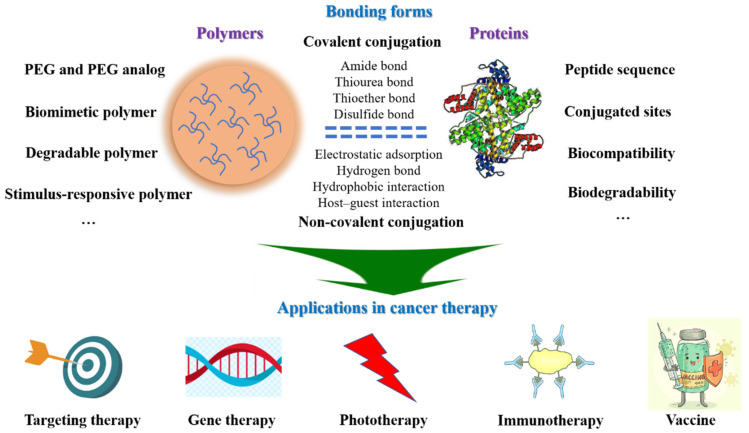
Overview of design and application of hybrid polymer–protein systems in cancer therapy.

Here, we summarize an overview of the design and application of hybrid polymer–protein systems in the field of cancer therapy (Figure 1). In this review, the design ideas and considerations of hybrid polymer–protein systems are overviewed. Along with the latest developments in cancer therapy, the applications of polymer–protein systems in targeted treatment, gene therapy, phototherapy, immunotherapy and vaccines are highlighted. Finally, the current challenges and future prospects for developing hybrid polymer–protein systems as cancer therapy platforms are discussed.

## 2. Design of Hybrid Polymer-Protein Systems

Polymer–protein systems show great potential for improving hydrophobic drug solubility, enhancing drug biological distribution and pharmacokinetics and providing priority accumulation of targets. When selecting polymers and proteins to form a system, full consideration should be given to their properties, functions, connections and applications.

### 2.1. Selection of Polymers

The selection of polymers with good properties can maximize the anticancer effect of hybrid polymer–protein systems. The first thing to consider is the size of the polymer. Although larger polymers generally increase the half life, larger sizes make it easier for them to gather, leading to decreased kidney clearance and creating obstacles to metabolism in the body. Therefore, the smallest molecular weight polymer that increases the circulating half-life of the protein to the level required for the intended treatment should be selected. Moreover, the size of the polymer has an effect on biological activity [17]. Larger polymers often produce conjugates with lower biological activity, most likely due to non-specific steric hindrance. The ability of polymers to bind to proteins is another factor in the hybrid system. Additionally, the chemical nature of polymers affects the overall architecture of the conjugate. The shape of the polymers or polymer topology affects the properties and functions of the system. Brush-like, hyperbranched or dendritic topologies often have good characteristics compared to their linear counterparts while forming polymer bioconjugates [18]. In addition, degradability, half life, immunogenicity and related toxicities of polymers should be considered.

PEG and PEG analog, biomimetic polymer, degradable polymer and stimuli-responsive polymer represent a group of polymers explored in polymer–protein conjugation. Common functional polymers are as follows. Several types of polymers used in hybrid polymer–protein systems are summarized in Table 1.

#### 2.1.1. Polyethylene Glycol (PEG) and PEG Analog

PEG, a linear and nonionic polyether diol with the molecular formula HO-(CH_2_-CH_2_-O)_n_-H, is widely used in the modification of hybrid biological polymers, such as proteins and peptides. PEG is one of the most commonly used polymer materials and an excipient in the pharmaceutical industry with many advantages of non-toxic, non-irritating and good water solubility. PEG modification can change the physical and chemical properties of drugs, including conformation, electrostatic binding, hydrophobicity, etc. These physical and chemical changes can increase the retention time of drugs in vivo and prolong absorption time. PEGylation shields antigenic epitopes through steric hindrance, thereby reducing the immunogenicity of the drugs. Additionally, steric hindrance shields the drugs from enzymatic degradation and opsonization with serum proteins. Drug binding affinity to cell receptors can also be affected by PEG modification, thereby reducing nonspecific uptake by the mononuclear phagocyte system and improving the tumor targeting ability of delivery systems [19,20]. On the other hand, PEG modification can reduce the frequency and incidence of adverse events administered as well as improve drug efficacy and tolerance [21]. In addition, PEG can also increase the solubility and stability of proteins which is also beneficial for the production and storage of drugs [22,23].

In earlier studies, the immunogenicity and antigenicity of PEG were not found, and PEG has been considered a biologically inert material. Since then, PEGylation has evolved dramatically as an important way to improve the pharmacokinetics and pharmacodynamics of protein drugs [24]. However, with more and more PEGylated protein drugs entering clinical applications, side effects caused by the administration of PEGylated protein drugs are gradually increasing. For example, PEGinesatide (Takeda, Deerfield, IL, USA) was withdrawn from the market due to severe hypersensitivity reactions with fatal consequences which may be related to PEG [25,26]. Many reports have claimed that the production of anti-PEG antibodies in patients and the loss of drug efficacy continue to appear [27,28,29].

**Figure 2:**
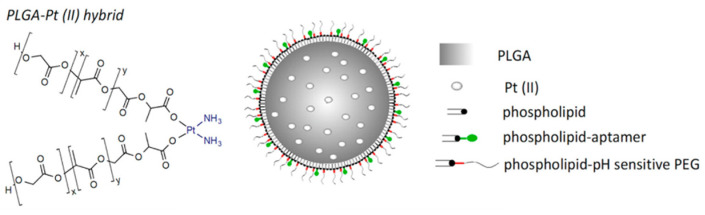
The proposal of the pH-MUC1-Pt A pH-sensitive PEG derivative polymer platform [35].

In order to solve these problems, PEG analogs were born on demand. PEG-like brush polymers not only enhance cycling half life but also do not induce an anti-PEG antibody response. For example, poly (polyethylene glycol methyl ether methacrylate)—p (PEGMA) is a methacrylate polymer having a low ethylene glycol side chain that has been used in a variety of protein–polymer conjugates [30,31]. In addition, poly(N-(2-hydroxypropyl) methacrylamide) (p(HPMA)) is another water-soluble and biocompatible polymer that can be used to increase half life in vivo and decrease associated immunogenicity [32,33]. Furthermore, zwitterionic poly(carboxybetaine) (PCB) with good biocompatibility and superior hydrophilicity was introduced as a PEG substitute for protein modification. As reported by Li et al. [34], the generation of PCB-specific antibodies was minimal and insensitive to increasing protein immunogenicity. A pH-sensitive PEG derivative polymer, poly(lactic-co-glycolic acid) (PLGA), coated with phospholipid-DNA aptamers and an acid-labile hydrazone linkage was designed (Figure 2) [35]. The hybrid polymer encapsulating platinum (II) could target a cell-spanning protein overexpressed to more than 90% of late-stage ovarian cancer, mucin 1 (MUC1), thus achieving enhanced cancer therapy. It follows that PEG analogs have a wider application prospect in the field of biomedicine.

#### 2.1.2. Biomimetic Polymer

**Figure 3:**
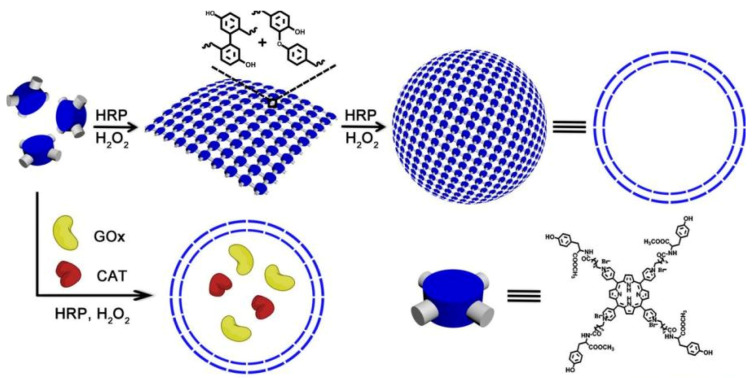
The formation of nanocapsules and GOx/CAT-NCs [49].

Biomimetic polymer is a kind of polymer that resembles natural polymers in form, appearance and properties with the benefits of synthetic polymers. For example, trehalose can form a special protective film in cells under harsh conditions, such as high temperature, high cold and dry water loss, effectively protecting the structure of biomolecules from being destroyed, thus maintaining the life process and biological characteristics of living organisms [36,37,38]. Therefore, natural disaccharide trehalose polymers can stabilize biological macromolecules under extreme conditions. For example, Diaz-Dussan et al. [39] reported a trehalose-based polymer that can be used as a cryoprotectant and three-dimensional (3D) cell scaffold for cell encapsulation and organoid production. Polyelectrolytes (PEs) molecules that carry multiple negative or positive charges may be functionalized by absorbing proteins at the top of the architecture or by inserting one or more kinds of proteins [40]. In particular, natural PEs have been extensively investigated for drug delivery because of their readily available, low toxicity, biodegradable and biocompatible properties. Among them, polysaccharides are the most promising materials, such as dextran [41], cellulose [42], and pectin [43], which are naturally derived linear and/or branched polymers widely used as PEG alternatives in the fabrication of therapeutic protein/drug conjugates. Recently, PSA-rFVIII (a novel polysialylated recombinant coagulation factor VIII—BAX 826) has completed a Phase I clinical trial (NCT02716194) in patients with severe hemophilia A and demonstrated long circulation half life with adequate safety and tolerability [44]. Another example of biomimetic polymers is heparin-mimicking polymers, such as carboxymethyl benzylamide sulfonate dextrans (CMDBS) [45], polysulfonated polymers [46,47] and sulfated synthetic glycopolymers [48]. Covalent dimerization of tyrosine in a horseradish peroxidase (HRP)-catalyzed system was an efficient method for enzyme-catalyzed preparation of polymers. Liu et al. constructed polymer nanocapsules with porphyrin photosensitizers as building blocks based on HRP-catalyzed tyrosine dimerization (Figure 3) [49]. The polymer nanocapsules further encapsulated glucose oxidase (GOx) and catalase (CAT) to obtain a biomimetic cascade nanoreactor (GOx/CAT-NC) for starvation and photodynamic cancer therapy which contribute to the advancement of complementary modes of spatiotemporal control of cancer therapy. These biomimetic polymers can stabilize, encapsulate and control release proteins, enhancing therapeutic efficacy and reducing side effects in therapeutic applications. 

#### 2.1.3. Degradable Polymer

Non-degradable polymers can accumulate in the body and may cause potential toxic side effects to the body, so it is necessary to develop degradable polymers for biomedical purposes [50]. Cyclic ketene acetal (CKA) is a monomer that produces hydrolyzable backbone ester bonds by a free radical ring-opening reaction [51]. CKAs can be copolymerized with vinyl monomers to produce functional aliphatic polyesters which are degradable polymers widely used in medical science [52,53,54]. In addition, there are some natural polymer analogs, such as peptides [55] and hydroxyethyl starch (HES) [56], which are degradable in vivo. Various proteins have been conjugated to synthetic polypeptides, and although these peptides are stable, they can be degraded in vivo by proteases. Remarkably, several micellar formulations based on degradable poly(ethylene glycol)-b-polypeptide block copolymers (NK105, NC6004, NK911, etc.) have been developed into different phases of clinical trials to treat breast, pancreatic and gastric cancers [57]. HES can be degraded by α-amylase in plasma, and its conjugates have been extensively studied for therapeutic applications [58,59,60]. PLGA is a common biodegradable polymer that is usually broken down in the body into LA and GA which is eventually metabolized by the body into carbon dioxide and water. Due to its outstanding biocompatibility, biodegradability and mechanical properties, PLGA has been approved by the FDA and the European Medicines Quality Agency (EMA) as a superior drug carrier. PLGA-based drug delivery systems effectively improve the bioavailability of drugs, reduce adverse effects and reduce the frequency of dosing which may improve patient compliance with medication [61]. Biodegradable polymers are both synthetic and natural, and all biodegradable polymers are basically stable and durable in applications. In terms of application, biodegradable polymers are of great significance in medical treatments.

#### 2.1.4. Stimulus-Responsive Polymer

**Figure 4:**
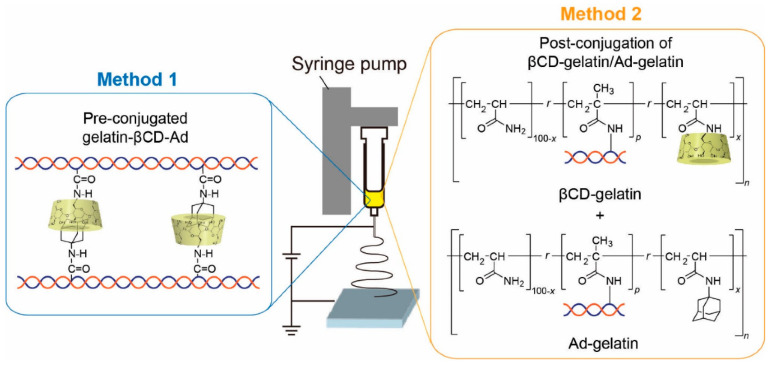
Overview of the fabrication of stimulus-responsive nanofibers [67].

In addition to improving pharmacokinetics, polymers can also impart new properties to proteins to create a stimulus-responsive system. For example, polyacrylamides, such as poly(N-isopropyl acrylamide) (p(NIPAAm)), have a lower critical dissolution temperature which allows polymers to precipitate with increasing temperature [62]. There are also conjugates of pH-responsive ionic polymers, such as polyacrylic acid [63] and poly (N,N-dimethylaminoethyl methacrylate) (p(DMAEMA)) [64]. The use of stimulus-responsive polymers in disease theranostic applications may enable a more controllable and effective process, for example, hyperthermia-mediated cancer treatment [65] and acid response nanoprobe in cancer diagnosis [66]. Polymer- and/or protein-based nanofibers that promote stable cell adhesion have drawn increasing attention as well-defined models of natural 3D extracellular matrix. Kentaro et al. fabricated two types of stimulus-responsive gelatin-containing supramolecular nanofibers that can be utilized as well-defined, switchable 3D microenvironments for cells. The first type of nanofibers was prepared by coupling the host–guest inclusion complex to gelatin before electrospinning, while the second type of nanofibers was fabricated by coupling gelatin to polyacrylamide functionalized with the host (βCD) and guest (Ad) moieties followed by conjugation in the electrospinning solution (Figure 4) [67]. The stimulus responsive was achieved by elasticity switching under physiological conditions by adding/removing soluble guest molecules. In addition to this, there are other polymers that respond to redox agents [68], photo irradiation, CO_2_ [69], enzyme [70], electric [71] and/or magnetic field [72], photo radiation [73] and so on.

**Table 1:** Several types of polymers used in hybrid polymer–protein systems.

Category	Example	References
PEG and PEG analog	p(HPMA)	[32,33]
	PCB	[34]
	Poly(lactic-co-glycolic acid) (PLGA)	[35]
Biomimetic polymer	Trehalose-based polymer	[39]
	Dextran, Cellulose, Pectin polymer	[41,42,43]
	Heparin-mimicking polymer	[45,46,47,48]
	Horseradish peroxidase (HRP)-catalyzed system	[49]
Degradable polymer	CKA based polymer	[51]
	Peptides	[55]
	HES	[56]
	PLGA	[61]
Stimulus-responsive polymer	p(NIPAAm)	[62]
	polyacrylic acid	[63]
	p(DMAEMA)	[64]
	Gelatin-containing supramolecular nanofibers	[67]

These are the main types of polymers above-mentioned. Table 1 shows some examples of different types of polymers.

### 2.2. Section of Proteins

In general, polymer–protein coupling faces more spatial and entropy barriers than small-molecule coupling reactions. Therefore, the commonly used coupling methods need to be highly efficient, and the selection of coupling chemistry also needs to consider site selectivity and the availability of residues on proteins. Site-selective coupling is necessary to maintain adequate biological activity. Typically, the conjugated site should be located away from the active site or binding motif to maximize protein activity. Therefore, it is important to select proteins that contain easily modified residues, such as lysine, cysteine and disulfide bonds [74,75,76]. It is worth noting that protein active sites must be predefined, and the binding motif should be away from the conjugation sites so as not to affect protein activity [77]. For protein denaturation, protein aggregation during the denaturation process must be strictly avoided. Because it is difficult to disaggregate protein agglomerates once they have precipitated, this reduces yields and hinders the protein purification process. In addition, biocompatibility and biodegradability by proteases, peptide sequence, various functionalities in specific positions and tunable transition also need to be considered for well-defined polymer–protein systems [78].

### 2.3. Bonding Form of Polymer-Protein Systems

Many active reactive groups are present in both polymers and proteins which allows them to bind in a variety of ways mainly by covalent and non-covalent conjugation.

#### 2.3.1. Covalent Conjugation

Reactive groups on polymers are electrophiles that react with nucleophilic groups of proteins, such as lysine, cysteine and tyrosine residues, typically at one end of the polymer chain. These common reaction groups include activated esters, carbonates, aldehydes, amine-reactive mercaptide reagents, Michael receptors, disulfide bond exchange, N-terminal coupling, tyrosine conjugation, etc.

Lysine is the most abundant amino acid on the surface of proteins and is often the first choice to attempt nonselective conjugation. Although lysine is not commonly used for site-selective conjugation, it is still very effective at loading conjugation due to its amino group. Lysine has a positively charged ε-amino group and the group requires a neutral to basic pH to have sufficient nucleophilicity (pKa~10.5). The pKa (6–8) of N-terminal amine is significantly lower than that of lysine, selective N-terminal modification is usually pH-dependent on the N-terminal α-amino group [79]. Common lysine coupling reactions involve the formation of stable amide bonds using a payload of active esters, e.g., o-succinimide reagents N-hydroxysuccinimide (NHS) [80,81]. Lysine reacting with maleimide [82] or halogenated acetamide [83] and coupling by isothiocyanate [84] to form stable thiourea bonds are also conjugation methods.

The high nucleophilicity of cysteine makes it easy to modify, but free cysteine is rare and is usually located in hydrophobic pockets. On the one hand, disulfide formation is a kind of cysteine modification. Cysteine is present in the form of disulfide bonds in antibodies and only free or reduced sulfhydryl groups (–SH) are available for reaction with thiol-reactive compounds, so reducing free sulfhydryl groups by using dithiothreitol, thioglycerol, or other sulfhydryl reducing reagents is necessary for the next step of the reaction [85,86,87]. On the other hand, alkylation formation by using carbonyl compounds to form thioethers can achieve cysteine modification [88]. Cysteine may also be modified by maleimide [89] or vinyl sulfone [90] functionalized groups by Michael addition at neutral pH.

Disulfide bonds are present in most proteins, playing an important role in maintaining spatial and resulting biological activity. They can be used as residue-specific binding sites. Tian et al. [91] prepared a multifunctional nanocarrier by anchoring transferrin to hollow mesoporous silica nanoparticles through disulfide bonds which can be resolved in the presence of glutathione, achieving redox response.

Although tyrosine has a low utilization rate, most proteins contain tyrosine residues with a natural abundance of about 3.2% [92], and it can be modified through nitrating agents. Several coupling methods, such as diazotization coupling [93] and 4-phenyl-3H-1,2,4-triazoline-3,5(4H)-dione (PTAD) coupling [94], have been developed. In addition, histidine [95], glutamine [96] and aldehyde [97] tag modifications are also considered important modification sites.

On a technical level, grafting synthesis of polymers to proteins can be achieved by using these three synthetic strategies: grafting to, grafting from and grafting through [98]. In brief, grafting to is the coupling of a pre-synthesized polymer with a biomolecule, where a pre-formed reactive polymer is conjugated to a protein using a backbone chain with functional groups that are randomly distributed along the chain. Graft copolymer formation originates from the coupling reaction between the functional backbone and reactive branch end groups. Grafting from is a technique involving monomers that are polymerized using an initiation reaction on the membrane surface, where the polymer chain is grown from a protein macroinitiator. The grafting from process involves in situ growth of a polymer from a biomolecule or alternatively synthesis of a biomacromolecule using a preformed polymer as the initiator [99]. Both strategies can be followed using protein-reactive RAFT agents [100,101]. For example, a water-soluble RAFT agent was conjugated to a model protein, bovine serum albumin (BSA), via its free thiol group at Cys-34 residue [102]. In addition, protein-reactive RAFT chain transfer agents contain either NHS or pentafluorophenyl ester moiety that can bind to lysine residues, and alternatively maleimide or pyridyl disulfide moiety for binding to cysteine residues [103]. The use of these two techniques is more frequent than grafting through, also known as the macromonomer method, which is one of the simpler ways to synthesize a graft polymer with well-defined side chains. Grafting through can polymerize biomolecule-containing monomers yielding bioconjugates with multiple biofunctional groups along the polymer backbone. For example, Dan et al. reported a well-controlled synthesis of molecular brushes through the grafting through method by aqueous ATRP, in which oligo- and poly(2-oxazoline) were used as macromonomers for polymerization [104].

#### 2.3.2. Non-Covalent Conjugation

Non-covalent conjugation methods mainly include electrostatic adsorption, hydrogen bonding, hydrophobic, host−guest interaction [105,106], etc. Electrostatic adsorption is a common form of connection between proteins and polymers. The charged region of proteins can greatly facilitate proteins’ interaction with other molecules or surfaces. Due to their hydrophilic nature, charged amino acids tend to be located on the outside of proteins, where they can interact with surfaces. In terms of surface chemistry, protein adsorption is a key phenomenon that describes the aggregation of these molecules outside the material. The ability of a protein to remain attached to a surface depends largely on material properties, such as surface energy, texture and relative charge distribution. The size and spatial structure of proteins also affect adsorption capacity [107]. Due to the greater number of contact points between amino acids and the surface of the material, larger proteins are more likely to adsorb and remain attached to the surface. The temperature and size of side chains in polymers also affect the strength of the adsorption force. Tan et al. [108] found that the affinity of the hIgG2 monoclonal antibody (mAb) with the negatively charged copolymer-modified surface was higher for adsorptions at 50 °C compared with 20 °C. Because at this higher temperature (50 °C), hydrophobic and electrostatic interactions were enhanced. In addition, the size of the side chains in the copolymer structure also influences the electrostatic interactions between the copolymer-modified surface and protein molecules. In addition, changes in electrostatic force will facilitate the adsorption/desorption process for controlled-release systems. Kwon et al. [109] reported the electrostatic interaction between human lysozyme and negatively charged albumin-heparin microspheres was affected by ion exchange. While changing the charge in the solution, an adsorption/desorption process was promoted, achieving a controlled release of positively charged polypeptides and proteins.

Hydrogen bonds and hydrophobic interactions are also widely found to hold proteins and polymers together. Hydrogen bonds can be defined as bonds formed between highly electronegative atoms, such as fluorine, oxygen, nitrogen, sometimes chlorine, etc. and hydrogen atoms. In contrast, hydrophobic interaction is a multifaceted phenomenon [110]. Briefly, non-polar molecules or groups of molecules attract each other and gather in the water environment, and the original water molecules near the non-polar groups are crowded out, resulting in an increase in the entropy of the surrounding water molecules. Hydrogen bonds and hydrophobic interactions often play a key role in the formation and self-assembly of polymer–protein hybrid systems. For example, polyamidoamine (PAMAM-G4) conjugated with serum albumin via hydrophobic and hydrogen-bonding interaction, achieving high protein loading efficacy (45–55%) [111]. Cai et al. [112] reported that hydrophobic interactions induced poly(N-isopropylacrylamide) (PNIPAM) and bovine serum albumin (BSA) to form polymer–protein hybrid self-assembly complexes in aqueous solution, while enhancing BSA stability (retaining over 90% of its activity). In addition, host–guest interaction is also commonly used in the formation of polymer–protein self-assembly complexes. For instance, a water-soluble pillar [5] arene and a lysine derivative were used for host–guest complexation, and the obtained complex was employed in the formation of drug-loaded vesicles for controllable drug release [113].

The main bonding forms of polymer–protein systems are summarized in Table 2.

### 2.4. Consideration in the Design Process

**Figure 5:**
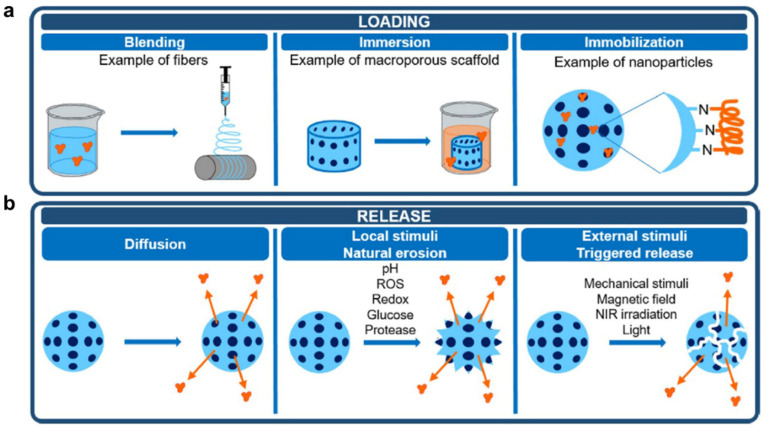
Protein loading (**a**) and release (**b**) methods [114].

For cancer treatment systems, proteins often play therapeutic or adjuvant therapeutic roles alone or in synergy with polymers. During the design process of anti-cancer systems, reasonable polymer–protein binding and release methods must be considered according to anti-cancer treatment strategies. A representation of protein loading and release methods is shown in Figure 5.

Commonly protein loading steps include (Figure 5a): (1) Polymers blend with protein in a solution at an appropriate temperature. In this process, the choice of temperature and solvent is important to ensure that proteins and polymers activity is not affected. (2) During immersion, proteins absorb to the surface and/or interior of polymers. This process can be assisted by stirring, so that proteins and polymers can contact more evenly. (3) The immobilization process allows proteins to bind to polymers by covalent bonds or non-covalent bonds mentioned above, forming ultimate systems. The entire loading process should ensure that protein activity is not affected by external conditions. Meanwhile, reaction solvents, temperature, pH, stirring speed, etc. can be controlled to prepare a complex system with suitable particle size and protein loading for subsequent treatment. In addition, the protein’s binding to the polymer is also crucial. The desirable linker or spacer must be selected to be adequately stable during circulation, protect the drug from premature metabolism and facilitate the release of drugs enzymatically or hydrolytically cleavage. In addition, other anti-cancer drugs or adjuvants can also be loaded at the same time during the protein loading process to achieve one-pot preparation. In another embodiment, proteins are added during polymerization to form polymer–protein complexes or polymer-coated protein systems, e.g., liposomes loaded with proteins.

In a polymer–protein system, proteins often need to be released for their function to develop. The release methods include (Figure 5b): (1) Proteins self-diffuse through the matrix, leading to desorption of the systems. This process is slow and difficult to release proteins completely. (2) Local stimuli, such as pH, redox, ROS, glucose and protease can contribute to the natural erosion of the system, promoting the release of proteins and other drugs. Especially for cancer therapy, the tumor microenvironment is characterized by low pH and high H_2_O_2_ levels which is ideal for responsive drugs to play their roles. (3) Some external stimuli, such as mechanical stimuli, visible or near-infrared (NIR) irradiation, magnetic field, etc. can induce the degradation of the system and reduce the binding forces between polymers and proteins, boosting the controlled release of proteins.

In order to obtain an ideal system, other details are also worth considering, such as biocompatibility of materials, the proteins’ density on the polymers, distance from the contact surface, the release rate and manner of proteins, the possible hazards of responsiveness, etc.

## 3. Applications for Hybrid Polymer-Protein Systems in Cancer Therapy

### 3.1. Targeted Treatment

Polymer–protein anticancer drugs always have their own tumor-targeting ability with high efficiency and low toxicity which can effectively avoid side effects. Research on targeted polymer–protein drugs has been a hot topic in recent years. Nowadays, targeted cancer therapies are mainly divided into two types: monoclonal antibodies targeting proteins on the surface of cancer cell and small molecule inhibitors targeting intracellular kinases and some other enzymes. Compared with small molecule drugs, protein drugs have the characteristics of high activity, high specificity, low toxicity and clear biological function and are conducive to clinical application.

#### 3.1.1. Tumor Cells Targeted

**Figure 6:**
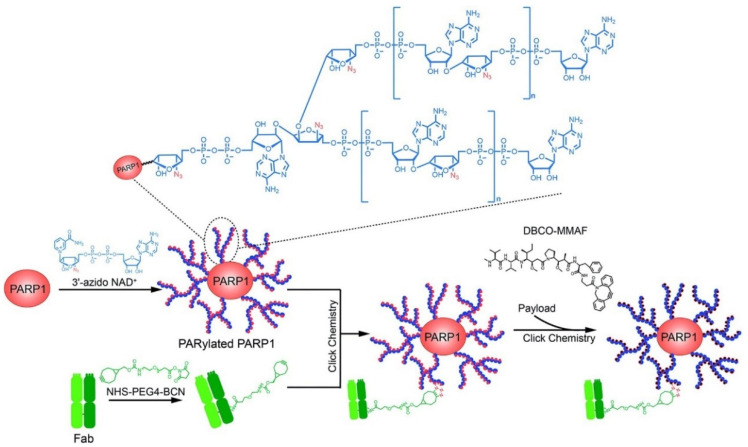
Schematic of the design and generation of a poly-ADP-ribose polymer-based antibody-drug conjugate [118].

Targeting tumor cells is the preferred strategy for tumor-targeted therapy. Receptors targeting tumor cell proteins tend to be highly expressed on the surface of tumor cells, such as epidermal growth factor receptor (EGFR), insulin-like growth factor receptor (IGF-IR) [115], folate receptor (FR) [116], gonadotropin-releasing hormone receptor (GnRHR) [117], etc. The carrier material is constructed by chemical modification of targeted peptides to target tumor cell surface receptors, and the complex drug system will execute direct targeting of tumor cells which can effectively improve the clinical treatment effect and safety of cytotoxic drugs, enhancing the patient’s adaptability to the drugs. Shi et al. [118] prepared complex polymers loading protein poly-ADP-ribosylated PARP1 with 3′-azido ADP-riboses, anti-human epidermal growth factor receptor 2 (HER2, a type of EGFR) antibodies and monomethyl auristatin F (MMAF) through covalent conjugation (Figure 6). The designed PARylated PARP1-antibody-MMAF conjugate could potentially kill HER2-expressing cancer cells in high specificity, potentially improving the physicochemical and pharmacological properties of cancer therapy.

#### 3.1.2. Tumor Stem Cells Targeted

**Figure 7:**
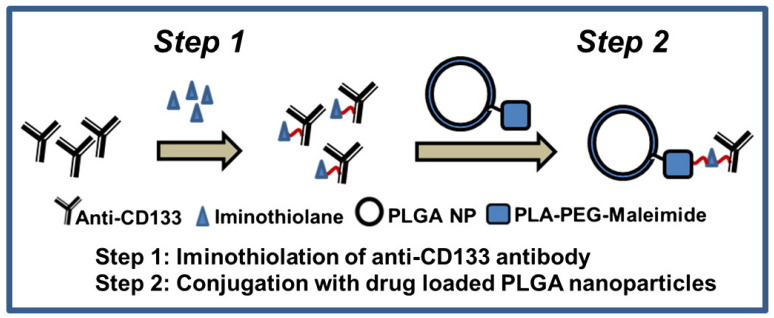
Anti-CD133 antibody or control IgG antibody was iminothiolated and then conjugated to maleimide functionalized PLGA nanoparticles [119].

Tumors are generally thought to be formed by mutations in somatic cells, and each tumor cell can grow without restriction. However, it does not make sense that tumor cells seem to have unlimited vitality and not all tumor cells can grow indefinitely. The characteristics of tumor cell growth, metastasis and recurrence are very similar to the basic characteristics of stem cells. Therefore, cancer stem cells are a group of cells in tumor cells with self-renewal capacity and are believed to cause cancer, promote tumor progression, metastasis and recurrence. Cancer stem cells are highly resistant to conventional treatments due to many factors, such as the differential surface antigens of tumor stem cells. For example, cancer stem cells have less surface antigen and are more difficult to destroy than normal tumor cells. Common surface markers of cancer stem cell include: CD133 (Prominin-1), CD44, stem cell antigen-1 (Sca-1), epithelial/endothelial cell adhesion molecule (EPCAM), sex-determining region Y-box protein 2 (SOX2), etc. Swaminathan et al. [119] developed polymeric nanoparticles targeting CD133 by conjugating an anti-CD133 monoclonal antibody to nanoparticles formulated using poly (D, L lactide-co-glycolide) (PLGA) polymers. Anti-CD133 antibody was iminothiolated and then conjugated to maleimide functionalized PLGA nanoparticles for breast cancer therapy (Figure 7). The polymer–protein systems loaded with paclitaxel lead to a significant reduction in tumor initiating cells and improved therapeutic efficacy compared to free paclitaxel therapy. The results indicate that anticancer therapeutics based on polymer and monoclonal antibody targeting CD133^+^ cancer stem cells could inhibit tumor growth and have the potential to prevent tumor recurrence.

In addition to the actively targeted antibodies described above, albumin can also target tumor cells. Because the rapid reproduction of tumor cells requires a large number of nutrients, albumin can provide amino acids and energy, so that albumin-binding receptor Gp60 exists on the surface of tumor cells. When albumin or albumin-modified polymers are used as delivery carriers, they can target the Gp60 receptor and then bind to intracellular protein (caveolin-1). The cell membrane is invaginated to form transport vesicles, so albumin carriers accumulate in the tumor to achieve targeted drug delivery. Due to their high content of charged amino acids, albumin-based polymer nanoparticles could allow electrostatic adsorption of positively (e.g., ganciclovir) or negatively charged (e.g., oligonucleotide) molecules [120]. It can also be combined with certain molecules (e.g., folic acid) to form an active targeting system. Therefore, albumin or albumin-based polymers are excellent transporters with their biocompatible, non-immunogenic and biodegradable properties that can form complexes with poorly soluble or exogenous substances, becoming the “ferry” of many substances in the blood circulation. In 2005, FDA approved paclitaxel albumin-bound nanoparticles (Abraxane®) for the treatment of metastatic breast cancer after the failure of combination chemotherapy, becoming the world’s first successful case of albumin nanoparticle drug delivery technology [121]. Liu et al. formed amphiphilic bioconjugates of BSA-poly(2-hydroxypropyl methacrylate) (BSA-PHPMA) for intracellular protein delivery [122]. Albumin polymer-based systems can also be used for targeted therapy for in situ gliomas. Yang et al. [123] designed an albumin based phototheranostic probe which could penetrate the blood−brain barrier (BBB), accumulating into deep-seated gliomas via albumin-binding protein mediated transportation.

#### 3.1.3. Tumor Microenvironment (TME) Targeted

In addition, TME targeting is also an important protocol. TME is mainly divided into two parts: tumor angiogenesis and tumor stromal cells. Angiogenesis provides energy for tumor growth and pathways for tumor metastasis. Therefore, inhibition of tumor angiogenesis has become an important means of treating cancer. Tumor vascular inhibitors are popular in the development of anti-tumor drugs at present, such as popular targets vascular endothelial growth factor (VEGF) or vascular endothelial growth factor receptor (VEGFR) family, platelet-derived growth factor (PDGF), angiopoietin 1/2 (Ang1/2), hepatocyte growth factor (HGF), galectin-3, etc. For example, molecularly imprinted polymer nanoparticles (nanoMIPs) with anti-VEGF antibodies were proved to specifically target VEGF and home to tumor mass in xenotransplantation of human malignant melanoma cells in zebrafish embryos [124]. VEGF signaling is considered the primary promotor of angiogenesis. In addition to angiogenesis control, these drugs can potentiate immune therapy because VEGF also exhibits immunosuppressive functions [125].

TME contains not only tumor cells and blood vessels but also stromal cells. For example, fibroblasts are the most important stromal cells and cancer-associated fibroblasts (CAFs), also known as activated fibroblasts or myogenic fibroblasts, form a physical barrier for tumors that protects them from multiple anti-tumor drugs. Feng et al. [126] developed a CAFs-targeting biodegradable polymer nanoparticle coated with CREKA peptide and loaded with traditional Chinese medicine (TCM) to inactive CAFs, reduced extracellular matrix production, promoted tumor vascular normalization and enhanced blood perfusion at pancreatic tumor sites. Therefore, the therapeutic strategy of targeting TME based on polymer–protein systems has become a promising idea for the treatment of tumors.

### 3.2. Gene Therapy

**Figure 8:**
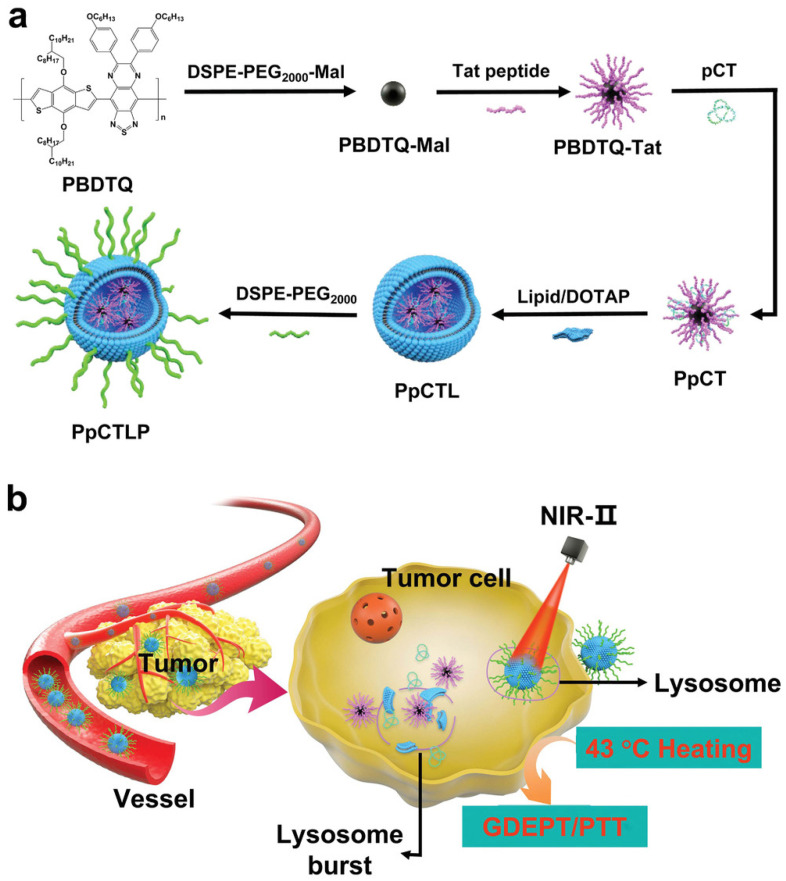
(**a**) Illustration of the preparation of PpCTLP NPs (PBDTQ/pCT/Lipid-PEG) and (**b**) the delivery and activation of PpCTLP for synergistic tumor GDEPT/PTT triggered by near-infrared-II (NIR-II) laser at mild hyperthermia [127].

Polymer–proteins often play the role of carriers in cancer gene therapy due to their excellent properties, such as biodegradablility, controlled release ability and good loading performance. As shown in Figure 8a, Zhang et al. [127] applied maleimide group decorated poly{[4,8-bis((2-octyldodecyl)oxy)benzo [1,2-b:4,5-b′]dithiophene-2,6-diyl]-alt-co-[6,7-bis(4-hexyloxy)phenyl]-[1,2,5]thiadiazolo [3,4-g]quinoxaline-4,9-diyl]} (PBDTQ) nanoparticles (PBDTQ NPs) to combine with the positively charged cell nucleus-targeting Tat peptide (YGRKKRRQRRRC) through chemical conjugation to form PBDTQ-Tat NPs which could enhance the ability in combining plasmids. Subsequently, the plasmid-expressing complex heat-inducible gene based on the HSP70 promoter (pDNA)-cytosine deaminase (CD)-cytosine deaminase (TK) (pCT) was combined with PBDTQ-Tat NPs via electrostatic interactions (PpCT). Finally, it is coated by cationic liposomes (PpCTL) to improve stability. Under NIR irradiation, due to local temperature rise, prodrugs were released and HSP70 promoters were active (Figure 8b). The polymer–protein systems are well-implemented stimuli-responsive cancer gene therapy with excellent specificity and safety.

Silk-elastin-like protein polymers (SELPs) have been effectively used as controlled-release matrices for the delivery of viruses for cancer gene therapy. Price et al. [128] used recombinant techniques to tune the degradability of these polymers for improved localized intratumoral gene delivery. SELPs were sensitive to specific matrix metalloproteinases (MMP). In addition, MMP-responsive SELPs for viral-mediated gene therapy of head and neck cancer exhibited tunable properties. Furthermore, the albumin-based polymers mentioned above also have a good ability to carry gene drugs. Human serum albumin (HSA)-protamine sulfate-DNA ternary particles have been successfully utilized as a nonviral gene delivery vehicle, where they combine a high transfection potential with a good biocompatibility, low cytotoxicity and efficient cell uptake [129].

### 3.3. Phototherapy

In recent years, phototherapy as a non-invasive tumor treatment method has attracted widespread attention. Phototherapy refers to irradiating the lesion area through a light source, especially a near-infrared light source, to stimulate phototherapy reagents to kill tumor cells with selectivity and hypotoxicity. Phototherapy mainly includes two forms: photodynamic therapy (PDT) and photothermal therapy (PTT). PDT irradiates safe and non-toxic photosensitive molecules in dark places with light to produce singlet oxygen (^1^O_2_) that has a killing effect on tumor cells [130]. PTT, on the other hand, uses a light source to irradiate photothermal conversion agents, generating heat to cause local hyperthermia at the lesion site to kill tumor cells [131]. Polymer–protein systems play a major role in carriers and catalytic for cancer phototherapy.

**Figure 9:**
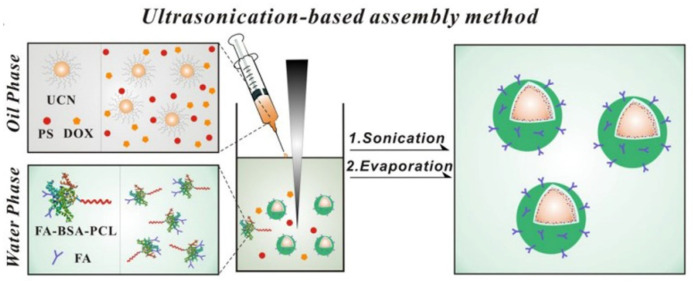
Fabrication of DOX-loaded UCN/ZnPc@FA-BSA-PCL nanosystem via an ultrasonication-based assembly method [135].

Photosensitizers and photothermal conversion agents are the main factors affecting phototherapy. Most photosensitizers are hydrophobic under physiological conditions; thus, they are easy to aggregate in solution, and their transports are affected in vivo [132,133]. Meanwhile, photosensitizers often exhibit short half life and non-targeting, so they have certain limitations in practical applications [134]. Photothermal agents face similar problems. Most photothermal agents are inorganic nanomaterials (e.g. gold nanoparticles) which often aggregate in solution as well. Polymer–protein systems pave the way to overcome these problems. For example, a multifunctional protein–polymer bioconjugate-coated upconversion nanosystem, consisting of upconversion nanoparticles (UCNs) core, a tailored amphiphilic bioconjugate protein-polymer shell, photosensitizer zinc phthalocyanine (ZnPc) and antitumor drug doxorubicin coloaded inside, was elaborately developed for combined PDT and chemotherapy (Figure 9) [135]. The protein-polymer bioconjugate with bovine serum albumin (BSA) and poly(ε-caprolactone) (PCL) by controllable maleimide–thiol reactions. The protein–polymer systems own excellent biocompatibility, low immunogenicity, low nonspecific protein adsorption and inherent biofunctional properties, while the polymer component as its tail imparts amphiphilic self-assembly properties, diversity, good stability and some other fascinating properties. Thus, they solved the hydrophobic problem of photosensitizers and improved the effect of combined therapy. Furthermore, HSA conjugated indocyanine green (ICG) (HSA-ICG) nanoparticles were developed by the programmed assembly, based on the intermolecular disulfide conjugations within HSA, for dual-modal imaging-guided cancer phototherapy [136]. Similarly, ICG was encapsulated by the amphiphilic polymer poly(styrene-co-maleic anhydride) (PSMA) to form ICG@PSMA nanoparticles [137]. Rong et al. [138] designed a NIR cyanine dye by introducing a rigid cyclohexenyl ring to the heptamethine chain to obtain a heptamethine dye CySCOOH as a photothermal conversion agent. HSA is a vehicle to transport CySCOOH via an ultrasonication-based assembly method, forming HSA@CySCOOH conjugates. The systems were applied to multi-modality NIR imaging-guided PTT with a low risk of dye leakage in the blood circulation and high dye loading efficiency.

The other major problem with PDT for antitumor application is the lack of oxygen at the tumor site which negatively affects the production of ^1^O_2_. Therefore, improving the tumor hypoxia microenvironment can boost the PDT effect. There are usually two ways to solve this problem. One is to supplement oxygen to tumor sites, and the other is to produce oxygen at tumor sites by enzyme catalysis.

**Figure 10:**
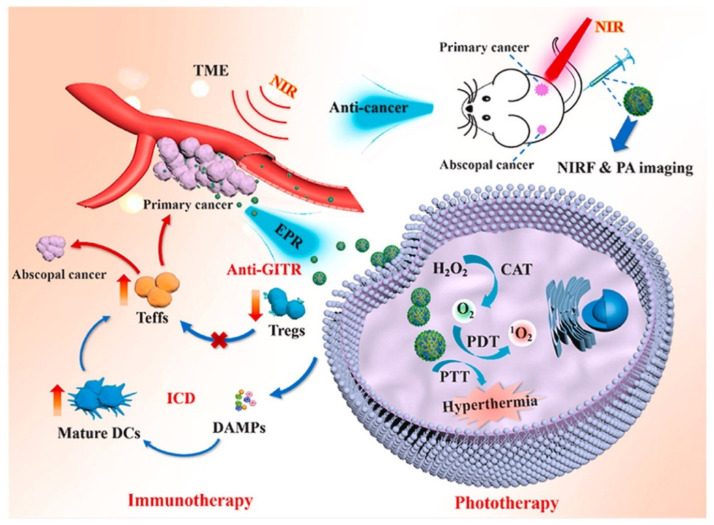
Schematic design of PDA-ICG@CAT-DTA-1 nanosystem possessing PTT, PDT, ICD effect, anti-GITR and imaging diagnosis functions based on both phototherapy and immunotherapy [140].

Hemoglobin (Hb) is a good first choice. Hb can achieve stable loading and maintenance of phototherapy agents (e.g., ICG) through electrostatic and hydrophobic interactions. Meanwhile, Hb can still maintain the ability to charge oxygen after loading phototherapy agents and provide oxygen during the PDT process to produce more ^1^O_2_ and improve the effect of PDT. Li et al. [139] designed ER-targeting pardaxin (FAL) peptides modified ICG conjugated-hollow gold nanospheres (FAL-ICG-HAuNS), together with oxygen-delivering Hb liposome (FAL-Hb lipo) to reverse hypoxia, achieving enhanced anti-tumor efficacy (Figure 10).

For the second solution, catalase (CAT) can play a good catalytic role in oxygen production. CAT catalyzes high levels of H_2_O_2_ at tumor sites to produce oxygen and water. However, free CAT becomes unstable in blood circulation due to the presence of proteases. Hence, CAT-based polymer-protein systems have been developed to protect CAT from protease digestion during circulation to enable efficient delivery to tumor sites. For example, a photo-based hypoxia-alleviated nanosystem was proposed to achieve enhanced phototherapy and immunotherapy for cancer [140]. CAT and anti-GITR antibody (DTA-1) were loaded to photothermal agent and photosensitizer composed polydopamine (PDA) conjugated ICG nanoparticles (PDA-ICG) via non-covalent connection, forming PDA-ICG@CAT-DTA-1 nanosystems. The PDA-ICG@CAT-DTA-1 exhibited intrinsic local hyperthermia and enhanced ^1^O_2_ generation in tumors and abrogated tumor immune suppression. Chen et al. [141] loaded CAT into its core by double emulsion method and decomposed H_2_O_2_ to produce O_2_ after being taken up by tumor cells. Zhang et al. [142] constructed a lipid-encapsulated CAT system which had a CAT loading rate of more than 10% and could still maintain more than 80% of the catalytic activity after 12 h of digestion by proteinase K.

### 3.4. Immunotherapy

At present, the field of immunotherapy is mainly focused on chimeric antigen receptor T cell immunotherapy (CAR-T) and checkpoint inhibitors (ICI), both of which aim to target cytotoxic T cells for the elimination of tumor cells. However, several problems reduce the therapeutic effect, including an immunosuppressive tumor microenvironment, a low level of drug infiltration into tumor tissues, off-target toxicity and other safety issues [143,144,145]. Furthermore, studies have shown that in order to achieve more powerful anti-tumor immune responses combined with immunotherapy, which can stimulate several different types of immune cells, needs to be considered [146,147]. This means that the drug delivery system needs to be loaded with multiple drugs. Polymer–protein nanosystems for cancer immunotherapy can serve as both immune adjuvants and delivery systems for immune-stimulating molecules.

**Figure 11:**
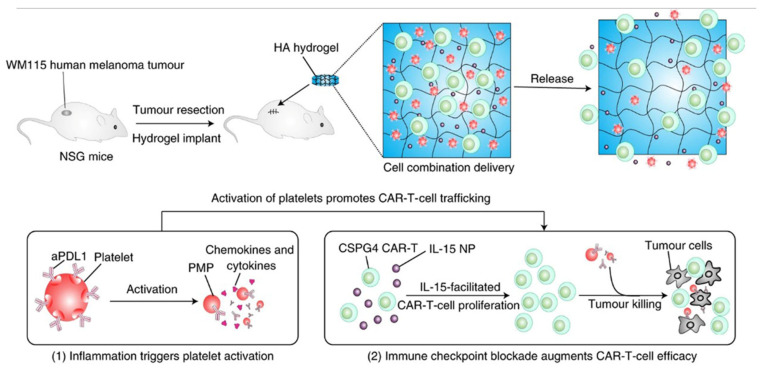
Schematic of the tumor resection model and implantation of the engineered HA hydrogel. Platelets activated during the wound healing process after surgery release aPDL1 in the form of PMP-aPDL1. MHC, major histocompatibility complex; TCR, T-cell receptor [150].

Cytokine–polymer conjugate systems can achieve immune-cell selectivity through polymer modification at key ligand/receptor interfaces. For example, Charych et al. [148] reported a prodrug Bempegaldesleukin (NKTR-214) which is a polymer-protein compound of PEG and interleukin-2 (IL-2). The IL2 core was bonded into the six (20 kDa) releasable PEG chains. Key hydrophobic and charged IL-2/IL-2Rα interaction sites were at or near sites of PEGylation. Thus, the PEG reagent and conjugation reaction could lead to facilitating localization on IL-2 at lysine residues clustered at the IL-2/IL-2Rα interface. Meanwhile, cytokine–polymer conjugates additionally with a hydrolysable polymer linkage could shed PEG at a controlled rate. In vivo, PEG chains could release to generate active IL2 conjugates slowly, thus prolonging IL-2 circulation and facilitating sustained cytokine delivery to T cells. Furthermore, the results showed that NKTR-214 enhanced persistence and antitumor immunity in combination with an anti-cytotoxic T lymphocyte-associated antigen-4 (CTLA-4) antibody in preclinical cancer models. Currently, NKTR-214 is in Phase III clinical trials in patients with skin cancer (NCT03635983), muscle-invasive bladder cancer (NCT04209114) and advanced metastatic renal cell carcinoma (NCT03729245). Similarly, Dapirolizumab pegol (Anti-CD40 ligand), NKTR-358, NKTR-262 and NKTR-255 polymer conjugates are also in different stages of clinical trials [149]. Hu et al. [150] developed a hyaluronic acid hydrogel encapsulated CAR-T cells targeting the human chondroitin sulfate proteoglycan 4 (CSPG4), the cytokine interleukin-15 (IL-15) nanoparticles and platelets conjugated with the checkpoint inhibitor programmed death-ligand 1 (CAR-T-P–aPDL1@gel) (Figure 11). The hydrogel was produced using acrylate-group-modified hyaluronic acid (HA). In addition, the results showed that HA hydrogel was an excellent reservoir for CAR-T cells, IL-15 nanoparticles and platelets carrying aPDL1. The hybrid hydrogels created a favorable milieu in which CAR-T cells eradicate residual tumor cells and prevent tumor recurrence.

In addition, the surface charge of chemokines can also be modulated to tune binding affinity to antibodies to form a hybrid protein polymer for enhanced cancer immunotherapy. Fang et al. [151] designed a protein which fuses CCL21 (6C-kine) mutein with a PD-L1-specific VHH binding domain by way of a flexible peptide linker. The chemokine-VHH fusion proteins had the abilities to improve targeted delivery and attract effector cells into the TME for improved immunotherapy.

### 3.5. Vaccines

Peptides/protein vaccines, nucleic acid vaccines and cellular vaccines are the main forms of cancer vaccines. Peptides/protein vaccines can be divided into two types. One is that it uses peptide/protein antibodies that are ubiquitous in a type of tumor, and these proteins can be injected directly or introduced into the lesions through certain microbial vectors to trigger an immune response. The other one is through isolating the antigen from patients’ bodies and then reintroducing the modified antigen that can trigger the immune system back into patients. Herein, polymer–protein nanoparticles have shown promising improvements as therapeutic cancer vaccines. Polymer–protein nanoparticles can avoid the rapid degradation of antigens and improve the stability of vaccine formulations. On the other hand, they provide good adjuvant performances and promote the activation of antigen-presenting cells (APCs). Meanwhile, the nanometer size enhances antigens enrichment in lymph nodes and further enhances their immune response ability [152].

**Figure 12:**
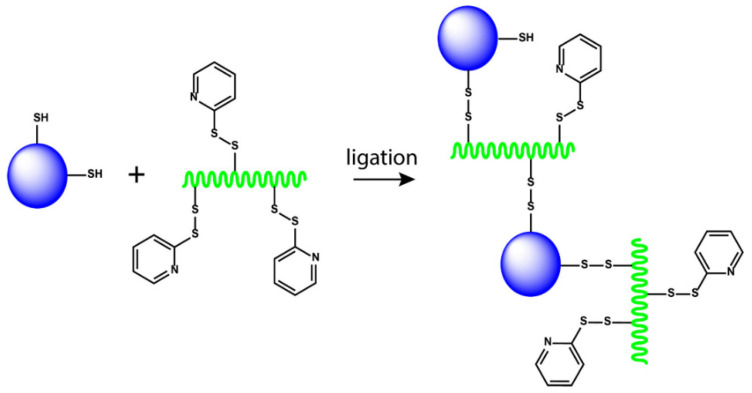
Schematic illustration of generic polymer–protein ligation strategy based on reversible disulfide formation between free thiols on a protein and pyridyldisulfide moieties on a polymer backbone. A mixture is obtained composed of multiple polymers per protein and/or multiple proteins per polymer [153].

**Figure 13:**
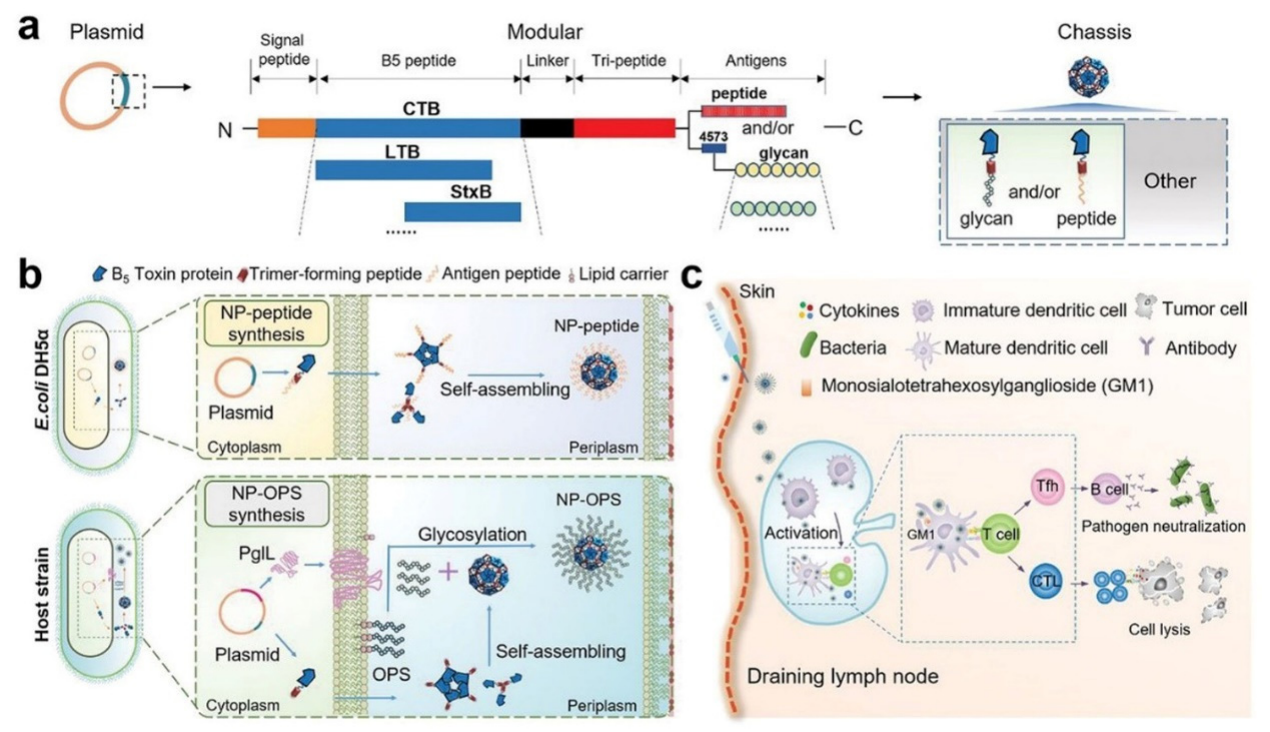
Design for the in vivo production of modular and self-assembling nanovaccines. (**a**) Modular design scheme of the nanovaccines and their versatile application configurations. (**b**) Cell-based fabrication of nanovaccines by expressing a fusion protein containing the B-subunit of AB5 toxin and a trimer-forming peptide in vivo. (**c**) Following vaccination, the nanovaccines quickly drain to the lymph node and activate APCs, leading to potent humoral and cellular immune responses [154].

For example, Lybaert et al. [153] reported a generic strategy for the formulation of antigens into nanoscale polymeric conjugates based on copolymers of N-hydroxypropyl methacrylamide (HPMA) with 3-aminopropyl methacrylamide (APMA) (Figure 12). The neutral hydrophilic HPMA moieties provide water solubility and biocompatibility. In addition, APMA units were used to introduce pyridyldisulfide moieties for reversible protein binding through disulfide formation as well as to enhance cell uptake through interaction with exofacial thiols. The water-soluble HPMA-based polymers with multiple pending pyridyldisulfide moieties were suitable for protein conjugation. In vitro experiments showed that the polymers could conjugate with ovalbumin (OVA), and the conjugation increased antigen presentation by APCs to CD8^+^ T cells. Pan et al. [154] developed a protein-based, self-assembling, stable nanovaccines bearing a variety of antigens, including peptides and polysaccharides nanovaccine platform (Nano-B5) (Figure 13a). Peptide and polysaccharide antigens were connected to the nanoparticle chassis in vivo via the expression of fusion domains and glycosylation by a bacterial O-oligosaccharyltransferase, respectively (Figure 13b). By efficiently draining lymph nodes after vaccination, antigens bearing on these nanovaccines could be well presented by APCs for T cell activation (Figure 13c). The results showed that the nanovaccines were safe in primates and mice and outperformed conventional vaccine preparations in stimulating a more efficient immune response which was a very promising cancer vaccine.

In addition, 3D printing technology combined with vaccine treatments is a new measure for cancer therapy which has greatly improved the treatment effect of cancer and improved the production efficiency of vaccines. Zhang et al. [155] used 3D-printed scaffolds loaded with immunoregulators for enhanced cancer immunotherapy. The new polymer–protein system with rapid manufacturing and precise molding based on 3D printing could enable mass manufacturing of cancer vaccines and personalized design.

Furthermore, due to the importance of innate immune co-stimulation in building a productive adaptive immune response, providing pathogen-associated molecular patterns (PAMPs) and/or damage-associated molecular patterns (DAMPs) that can bind pattern recognition receptors (PRRs) simultaneously with antigen administration to induce immunogenic cell death (ICD) is also necessary for cancer vaccines [156]. A dendritic cell (DC) vaccine based on α-lactalbumin (α-LA)-engineered breast cancer-derived exosomes with ICD inducers human neutrophil elastase (ELANE) and Hiltonol (TLR3 agonist) (HELA-Exos) was exerted by Huang et al. [157]. They found that HELA-Exos possessed a profound ability to specifically induce ICD and increase CD8^+^ T cell responses in breast cancer cells. This is a potential cancer vaccine stratagem for tumor inhibition in poorly immunogenic triple-negative breast cancer.

Herein, a list of hybrid polymer–protein systems with their applications for cancer treatments is summarized in Table 3.

## 4. Conclusions and Perspectives

As can be seen from this review, many cancer therapy design schemes and applications for hybrid polymer–protein systems have been developed to date. The selection of vital components, including polymers and proteins according to application purposes is crucial. The rationality of design schemes depends on the type of connections, reaction condition and protein release strategies which may influence the effect of subsequent application in cancer therapy.

Polymer–protein systems have the characteristics of high activity, high specificity, low toxicity, long circulation and some other clear biological functions which are ideal as vehicles or drugs for cancer therapy. Over the past decades, polymer–protein systems have been widely used in targeted therapy, gene therapy, phototherapy, immunotherapy and vaccine therapy for cancer, leading to tremendous progress. However, there are still challenges for hybrid polymer–protein systems in cancer therapy: (1) Nanomaterials need to be carefully selected to avoid toxicity in vivo. The size, shape, chemical composition, surface properties, agglomeration and/or aggregation status, and biodegradability and biocompatibility of hybrid polymer–protein systems may affect their toxicity in vivo which should be heavily considered in the prescription design and optimization processes. (2) Covalent or non-covalent modifications, as well as the application of organic solvents, can all change the structure of proteins. Therefore, in the preparation process, protein conformation and activity should be detected so as to verify the feasibility of the method and screen out the best preparation scheme. (3) The biotransformation and metabolic processes of hybrid polymer–protein systems in the body are complex and unclear. (4) Drug release processes of hybrid delivery systems in the body are difficult to control. Designing controlled release systems is an efficient and feasible method to solve the release problem. Characteristics of different parts of the body, such as pH, temperature, oxidation-reduction quality, etc., and external factors, such as photo, ultrasound and electromagnetism are considerable directions for controlled hybrid polymer–protein systems. (5) The biological activities of protein drugs before and after binding to targets need to be further confirmed. Although the complex structure of peptides and proteins enhances their potency and selectivity, it also results in their poor stability. They degrade easily under environmental storage conditions and are sensitive in vivo to pervasive protease, physiological temperature, and pH changes. To overcome these challenges, synthetic or humanized peptide analogs combined with unnatural amino acids or conjugated with known chemical components can improve half-life, stability, receptor affinity and reduce toxicity. (6) The mechanism of action of polymer–protein conjugates in vivo is complex. Polymer–protein conjugates may adhere to and/or embed in cell membranes or may be internalized into cells. There are various endocytosis pathways for hybrid biomaterials to enter cells, including clathrin-dependent, caveolin-dependent, non-clathrin and non-caveolin-dependent endocytosis, micropinocytosis and phagocytosis. At the cellular level, the agglomeration state can alter the size-dependent cellular internalization pathways and degree of uptake of biomaterials. In addition, circulating biomaterials regardless of exposure routes may also cross the blood–brain barrier, blood–testis barrier and placental barrier to reach the central nervous system, reproduction system and offspring, respectively, and have biological effects on organs [158]. (7) The establishment of excellent organoids, tumor models, especially humanized models and personalized treatment plans are challenging to address. New technology, for example, 3D bio-printed tumor models can be potential candidates for understanding drug action against tumors. At present, individualized clinical therapy relies primarily on therapeutic drug monitoring (TDM) and genetic testing. The accuracy and repeatability of existing detection methods should be ensured. On the other hand, to cater to the requirements of personalized medical care, speed up the advancement of novel diagnostic tools, create innovative approaches and equipment and enhance the efficiency of current methods, it is necessary to tackle this. (8) Some polymer–protein systems may face challenges in being applied in clinical settings due to their complex preparation procedures and expensive production costs. A comprehensive and succinct design methodology is required to effectively convey the clinical translation of polymer–protein systems. Meanwhile, pharmaceutical ingredients must be affordable and easily accessible while still meeting therapeutic demands. Additionally, the manufacturing process should be as convenient and feasible as possible for industrial production. (9) Regulatory requirements for the approval of polymer–protein systems designed in this manner present additional challenges. It is worth noting that, such as the drug design procedure, the regulation of polymer–protein systems submission follows the requirements of developing new chemical entities (NCEs). Similar to the regulatory requirements for peptide drugs, the developed polymer–protein systems must undergo thorough characterization for their structural, conformational, physiological and biological reactivity attributes. Prior to regulatory approvals, it is crucial to determine the quality, safety and efficacy profiles of these polymeric constructs. We anticipate that these challenges will progressively diminish in the foreseeable future, allowing for polymer–protein systems to remain a captivating domain utilized in cancer treatment and other diverse applications.

## Figures and Tables

**Table 2:** Bonding forms of polymer-protein systems.

Category	Example	References
Covalent conjugation	Amide bond	[80,81]
	Thiourea bond	[84]
	Thioether bond	[89]
	Disulfide bond	[91]
Non-covalent conjugation	Electrostatic adsorption	[108,109]
	Hydrogen bond	[111]
	Hydrophobic interaction	[112]
	Host–guest interaction	[113]

**Table 3:** Applications for hybrid polymer–protein systems in cancer therapy.

Category	Polymer	Protein	Reference
Targeted therapy	poly-ADP-ribose polymer	Anti-HER2 antibody	[118]
	PLGA	anti-CD133 antibody	[119]
Gene therapy	PBDTQ nanoparticles	Tat peptide, CD, TK	[127]
	Silk-elastinlike protein polymers	MMP	[128]
Phototherapy	Upconversion nanoparticles	BSA	[135]
	Gold nanospheres	Hb, pardaxin peptides	[139]
	PDA nanoparticles	CAT and DTA-1	[140]
Immunotherapy	PEG	IL-2	[148]
	Hyaluronic acid hydrogel	CSPG4, IL-15, aPDL1	[150]
Vaccines	Polymeric conjugates based on HPMA	OVA	[153]
	3D printed scaffolds	Antigen	[155]
	DC derived exosomes	Human neutrophil elastase, TLR3 agonist	[157]

## Data Availability

Not applicable.
